# Editorial: Mycobacterial dormancy, culturability, and resuscitation: state-of-the-art, challenges, and future prospects

**DOI:** 10.3389/fcimb.2023.1201012

**Published:** 2023-04-24

**Authors:** Elena G. Salina, Tatyana L. Azhikina, Bavesh Kana

**Affiliations:** ^1^ Bach Institute of Biochemistry, Research Center of Biotechnology, Russian Academy of Sciences, Moscow, Russia; ^2^ Shemyakin-Ovchinnikov Institute of Bioorganic Chemistry, Russian Academy of Sciences, Moscow, Russia; ^3^ Department of Science and Innovation/National Research Foundation Centre of Excellence for Biomedical Tuberculosis Research, School of Pathology, Infectious Diseases and Oncology Research Institute, Faculty of Health Sciences, University of the Witwatersrand and the National Health Laboratory Service, Johannesburg, South Africa

**Keywords:** persistence, resuscitation, mycobacteria, dormancy, non-replicative state

Mycobacterial pathogens cause a variety of diseases such as tuberculosis (TB), Buruli ulcer and leprosy, with the success of these pathogens primarily being driven by a plethora of mechanisms that facilitate escape from host immunity, ultimately leading to disease progression. In addition, mycobacteria can enter non-replicative states that limit their susceptibility to drug treatment, enhance immune subversion and affect recovery of bacteria on routine laboratory media (Batyrshina and Schwartz, 2019). With TB, the combination of these adaptations has driven a global epidemic of staggering proportion, primarily affecting the poor and placing strain on healthcare systems in resource limited settings. This situation has worsened due to the Covid-19 pandemic (WHO, 2022). The complexity associated with clinical presentation of TB, manifesting as asymptomatic infection, subclinical disease, incipient TB or active disease, has long been associated with altered metabolic states of the prevailing tubercle bacilli, but definitive evidence for these associations remains somewhat lacking (Lipworth et al., 2016; Drain et al., 2018; Behr et al., 2021). Tackling this, and appending issues, emerges as an important global priority to enhance diagnostic pickup, tailor prophylaxis approaches, shorten chemotherapy and reduce recurrent disease. This research topic, “Mycobacterial Dormancy, Culturability, and Resuscitation: State-of-the-art, Challenges, and Future Prospects” broadly focuses on mycobacterial adaptation to the non-replicative state, and subsequent resuscitation, together with efforts to identify molecular mechanisms related to these phenomena. Also included are studies aimed at developing novel therapies against drug tolerant bacteria. These, and other studies, are summarized in this editorial.

As model systems to replicate host conditions encountered by tubercle bacilli during pathogenesis continue to evolve, the review by Parbhoo et al. details adaptive strategies employed by *M. tuberculosis* to sense and coordinate physiological responses following exposure to various host-associated stressors that induce persister formation. The authors focus on exploring animal models for investigating different aspects of the immune response and the impact of the host environment, together with bacterial adaptation in contributing to recalcitrance of infection. Building on some of these aspects, their research article identified the importance of pathogen recognition, phagocytosis, phagosome acidification and maturation as host strategies in inhibiting intracellular growth of *M. tuberculosis*, with important consequences for persister formation. Parbhoo et al. studied how these host factors could impact intracellular bacterial burden, adaptive mechanisms and persistence in macrophages. Their findings provide important information that will direct future strategies targeting persisters to enhance bacterial clearance.

In the last decade, pathways involved in energy metabolism in mycobacteria have gained prominence as tractable areas for TB drug discovery. Plocinska et al. investigated the intracellular function of the Rv3143 protein, which influences the efficiency of the respiratory chain in mycobacteria and controls the switch to nitrate respiration, which is crucial at various stages of infection. Although the precise mechanism of action of Rv3143 requires further detailed analysis, this protein was identified as a component of the proton-pumping type I dehydrogenase complex (NDH-1) possibly modulating its activity.

Shifting to exploring molecular mechanisms that underpin mycobacterial dormancy, the review by Verma et al. focuses on detailing gaps in the understanding of mechanisms that are important for bacterial transition from dormancy to the replicative state and vice versa. Although several bacterial proteins have been identified as playing a key role in dormancy from a variety of studies using different models, how this information can be used to develop next generation therapies and vaccines is unclear. The review highlights novel drug candidates that are potent against non-replicating tubercle bacilli and discusses the potential of these agents to shorten the duration of treatment.

Characteristics of new vaccines and host factors that play an important role in modulating bacterial growth are also discussed. Extending on this idea, Sharma et al. identified phytomolecules that were specifically screened against dormant *M. smegmatis* obtained after prolonged incubation under low oxygen, low pH and nutrient limitation. The authors performed an in silico analysis focused on identifying the modes of action of these hits and found that some of them potentially target well known proteins/regulators involved in dormancy or cell cycle control.

The ability of *M. tuberculosis* to adopt non-culturable states, akin to those seen in laboratory models of dormancy, has important implications for the diagnosis of TB and mapping of transmission events. In this regard, the article by Chengalroyen et al. highlights this problem through detection of a heterogenous population of Differentially Culturable Tubercle Bacilli (DCTB) in sputum specimens from TB infected individuals. DCTB do not recover in routine laboratory media and this differential detection can influence diagnostic outcome and treatment regimens. The authors demonstrate that distinct *M. tuberculosis* lineages emerge differentially in liquid limiting dilution DCTB assays. These assays were supplemented with culture filtrate derived from *M. tuberculosis* as a source of growth stimulatory molecules. Their findings suggest that routine culture likely misses a large proportion of mixed TB infections, with important implications for selection of treatment regimens and in general, for key aspects of TB epidemiology such as transmission mapping. Related to this, Beltran et al. found that supplementation of cultures, with culture filtrate, from extrapulmonary clinical specimens can substantially improve culture positivity rates, with benefits for the diagnosis of spinal tuberculosis, which is usually paucibacillary nature and difficult to detect with routine approaches.

Diabetes is now considered an important risk factor for TB disease. Verma et al. studied the immunological cross-talk between diabetes and TB using the murine model of TB infection, with low bacterial loads in chronic infection to best mimic latent infection in humans. After establishing infection, diabetes was induced in a subset of animals using multiple doses of streptozotocin, followed by assessment of gene expression and cytokine production. The authors found that diabetes in TB infected animals led to a decrease in levels of monocyte chemotactic protein-1 (MCP-1), together with increased expression of matrix metalloprotease-1 (*mmp-1*), and a reduction in expression of *mmp-9*. These combined effects likely affect granulomatous containment of bacilli.

The search for master regulators that enable mycobacteria to respond to environmental stresses that trigger non-replicating persistence, such as hypoxia, has identified the DosR (dormancy regulon regulator), together with DosS and DosT as sensors (Sivaramakrishnan and De Montellano, 2013). In this article collection, Simcox et al. explore the function of DosR in the opportunistic pathogen *Mycobacterium abscessus* that is able to establish chronic infection in cystic fibrosis patients. The authors demonstrate that this regulatory circuit appears to coordinate a much larger set of genes (>1000) than those described in *M. tuberculosis*. Deletion of the DosRS homologues in *M. abscessus* led to attenuated growth of bacilli under low oxygen conditions, a shift from smooth to rough colony morphotype, and the down-regulation of numerous genes, thus providing the first outlook at the global transcriptomic response of this important pathogen.

Together, this research topic highlights important new data, providing a broad and diverse overview of challenges and prospects in area mycobacterial dormancy, culturability, and resuscitation. The Research Topic will be of interest to a wide audience, including researchers, physicians and clinicians, who study *M. tuberculosis* and other mycobacterial species that cause clinically relevant diseases.

## Author contributions

All authors listed have made a substantial, direct, and intellectual contribution to the work and approved it for publication.
